# Factors Associated with Language Delay in 12-Month-to-3-Year-Old Children—A Real-World Vietnam Case–Control Study

**DOI:** 10.3390/life16071050

**Published:** 2026-06-24

**Authors:** Thanh-Nhan Doan, Bao Thy Vuong, Thi-Linh-Giang Phan, Li-Wei Chou

**Affiliations:** 1 Department of Rehabilitation, Vinmec Da Nang, Vinmec International Hospital, Da Nang 11622, Vietnam; drchannhan@gmail.com; 2 College of Health Sciences, VinUniversity, Hanoi 100000, Vietnam; 3 Faculty of Health Sciences, Mekong University, Vinh Long 85000, Vietnam; vuongbaothy@mku.edu.vn; 4 Department of Rehabilitation, Quang Nam Northern Mountainous Region General Hospital, Da Nang 560000, Vietnam; phanlinhgiang070400@gmail.com; 5 Department of Physical Medicine and Rehabilitation, China Medical University Hospital, Taichung 404332, Taiwan; 6 Department of Physical Therapy and Graduate Institute of Rehabilitation Science, China Medical University, Taichung 406040, Taiwan; 7 Department of Physical Medicine and Rehabilitation, Asia University Hospital, Asia University, Taichung 413505, Taiwan

**Keywords:** language delay, risk factors, children, bilingual learning, rehabilitation

## Abstract

Objective: Language delay (LD) is a common developmental condition in which children fail to achieve age-appropriate language milestones, affecting communication, cognition, and social integration. It affects approximately 1 in 14 preschool children and may have long-term consequences into adulthood. The period from 12 to 36 months is a critical window for language development, during which children begin to comprehend and produce their first words. Early identification of risk factors during this stage is essential for timely intervention. However, in Vietnam, data on factors associated with language delay in this age group remain limited. Therefore, this study aimed to identify factors associated with language delay in children aged 12–36 months. Methods: A case–control study was conducted, including 55 children with language delay and 55 typically developing children aged 12–36 months. Personal, familial, medical, and environmental data were collected using structured questionnaires. Univariate and multivariable logistic regression analyses were performed to identify factors associated with language delay. Results: A total of 110 children (43 boys and 67 girls) were included. The strongest risk factor was the use of screens to calm or occupy children (OR = 36.6; *p* < 0.001). Early bilingual exposure was a significant protective factor (OR = 0.12; *p* = 0.014), while shared reading or picture viewing showed a strong but borderline protective effect (OR = 0.23; *p* = 0.051). Conclusions: The use of screens to calm or occupy children was the main risk factor for language delay, whereas early bilingual exposure and shared reading or picture viewing were protective factors. These findings highlight the importance of limiting non-interactive screen use and promoting interactive language activities to support early language development.

## 1. Introduction

Language development is a complex process that plays a crucial role in a child’s cognitive, emotional, and social growth. When a child fails to achieve age-appropriate language milestones, the condition is referred to as language delay (LD) [1]. LD is a common neurodevelopmental condition, affecting approximately 7–10% of children, even in the absence of intellectual disabilities, hearing impairments, or other developmental disorders [2]. Children with LD often have difficulty understanding and using language, expressing their thoughts, and maintaining social interactions. Without early detection and intervention, LD can lead to long-term consequences for learning, behavior, and social integration [2]. Language development encompasses both receptive language (the ability to understand spoken language) and expressive language (the ability to communicate thoughts, needs, and ideas through speech and other symbolic systems). Early language skills provide the foundation for later cognitive, academic, and social development. Delays in either receptive or expressive language may adversely affect communication, learning, and social participation throughout childhood [2]. Therefore, identifying early risk factors for language delay in children aged 12 to 36 months is essential to guide timely intervention and prevention strategies.

Globally, numerous studies have identified factors associated with language delay in young children. Language development is influenced by a complex interaction of biological, familial, socioeconomic, and environmental factors. Previous studies have identified several potential risk factors, including male sex, preterm birth, low birth weight, lower parental education, adverse socioeconomic conditions, and limited caregiver–child interaction. A recent scoping review of 12 studies, selected from 1894 articles across international databases, highlighted excessive screen exposure, child gender, number of siblings, family interaction, housing conditions, and parental socioeconomic status as important correlates of language delay [3]. Similarly, a large-scale study conducted in Thailand found that screen exposure exceeding two hours per day and a moderate-to-difficult temperament were significantly associated with suspected language delay [4]. However, findings regarding several biological and socioeconomic factors remain inconsistent across populations. While some studies have reported associations between language delay and demographic characteristics, others have suggested that environmental and interaction-based factors may play a more prominent role during early childhood. Consequently, increasing attention has been directed toward potentially modifiable factors such as screen exposure, caregiver–child interaction, shared reading activities, and language exposure patterns.

In contrast to risk factors, several protective factors have been reported to support early language development. Shared reading and picture-book activities provide opportunities for joint attention, vocabulary enrichment, and reciprocal communication between caregivers and children. Likewise, frequent verbal interaction and language-rich home environments have been associated with improved language outcomes [5]. Previous studies have also highlighted the importance of caregiver-directed speech and responsive verbal interactions in supporting early language development and the acquisition of early communication skills [6]. The role of bilingual exposure remains debated. Although some studies have reported temporary delays in vocabulary development within a single language, growing evidence suggests that bilingual children often achieve comparable overall language abilities and may benefit from enhanced cognitive flexibility and attentional control [7,8]. These findings highlight the importance of examining both risk and protective factors when investigating language development.

The Social Interactionist Theory proposes that language develops through reciprocal interactions between children and caregivers. Factors that promote meaningful communication, such as shared reading and verbal engagement, may facilitate language development, whereas factors that reduce opportunities for interaction, such as excessive screen use, may hinder language acquisition [9]. This framework informed the selection of several environmental and behavioral factors examined in the present study.

Despite increasing awareness of language delay in Vietnam, research in this field remains limited. Existing Vietnamese studies have primarily focused on prevalence estimates and screening outcomes, whereas relatively few investigations have examined factors associated with language delay in young children. Furthermore, there is a lack of standardized assessment tools and limited evidence regarding modifiable environmental and behavioral factors that may influence language development in Vietnamese children. These knowledge gaps are particularly important during the period of 12–36 months, which represents a critical stage of language acquisition and neurodevelopment.

Therefore, the present study aimed to identify factors associated with language delay among Vietnamese children aged 12–36 months. Based on previous literature, we hypothesized that screen-related factors would be associated with increased odds of language delay, whereas interaction-based factors, including shared reading and early bilingual exposure, would be associated with reduced odds of language delay.

## 2. Materials and Methods

### 2.1. Study Design

This age-matched case–control study was conducted at the Speech and Language Therapy Unit of the Department of Rehabilitation, Quang Nam Northern Mountainous Region General Hospital, Vietnam, between June and November 2025. The study aimed to identify factors associated with language delay among children aged 12–36 months. Children with language delay were recruited consecutively from the Speech and Language Therapy Unit during the study period. For each case, one age-matched control child (±3 months) with typical language development was recruited from the same residential area. Information on demographic, perinatal, environmental, family-related, and parenting-related factors was collected through structured interviews with parents or primary caregivers. All parents or legal guardians provided written informed consent prior to participation.

### 2.2. Sample Size Calculation

The sample size was calculated for a case–control study comparing two independent proportions. With a significance level of α = 0.05, corresponding to Zα/2 = 1.96, and a statistical power of 80%, corresponding to Zβ = 0.84, the expected exposure proportions were set at p1 = 0.587 for the case group and p0 = 0.286 for the control group, based on a previous [10]. The estimated minimum sample size was 47 participants per group. After allowing for a 10% rate of incomplete data, the required sample size was increased to 52 participants per group. In the present study, 55 children with language delay and 55 typically developing controls were recruited.

### 2.3. Participants

Inclusion criteria: Children aged 12–36 months whose parents or legal guardians provided consent to participate and who had complete information on medical history and environmental factors.

Exclusion criteria: Children with hearing or visual impairments, severe organic diseases requiring inpatient treatment, neurological disorders, or impaired consciousness were excluded. Parents or guardians who were uncooperative, unable to complete interviews due to language barriers or low educational level, or who had neurological, psychiatric, or serious physical illnesses were also excluded. Parents who were unable to provide reliable information during the interview, including those with difficulty communicating in Vietnamese, were excluded. Case group: The case group included 55 children aged 12–36 months diagnosed with language delay based on age-appropriate language development assessment criteria using the Rossetti Infant-Toddler Language Scale [11].

Language development was assessed using a Vietnamese-translated version of the Rossetti Infant-Toddler Language Scale (RITLS), which is routinely used in clinical practice at our institution. Language delay was primarily diagnosed by a trained physician and certified speech-language therapists based on clinical evaluation and age-appropriate developmental criteria. The RITLS was used to determine developmental language age and to support assessment in cases where the degree of delay required further clarification. When the diagnosis was equivocal, an operational criterion based on the degree of delay relative to chronological age (CA) was applied. The percentage of language delay was calculated as follows:

$$\%\;\mathrm{delay}\;=\;(1-\frac{\mathrm{DA}}{\mathrm{CA}}\;\times \;100)$$

DA (Developmental Age): language developmental age determined by RITLS assessment.

CA (Chronological Age): actual age of the child in months.

Children were classified as having language delay when the degree of delay was ≥33% (2SD) relative to their chronological age [12].

The cases were consecutively recruited from the Speech and Language Therapy Unit at the Department of Rehabilitation, Quang Nam Northern Mountainous Region General Hospital, between June and November 2025.

Control group: 55 children with typical language development. To ensure comparability, controls were recruited from the same residential areas as the cases and were individually matched to each case by age (within ±3 months). In practice, parents of children in the case group were asked to help identify potential control children from their acquaintances who met the inclusion criteria. After receiving referrals, potential controls were screened via telephone interviews to confirm typical development. Eligible children were subsequently provided with the study questionnaire and written informed consent forms. In cases where developmental status was uncertain based on the telephone screening, the child and their parents were invited to the hospital for direct assessment by a researcher to confirm normal language development prior to enrollment.

### 2.4. Data Collection

Data were collected using a structured questionnaire administered through interviews with parents or primary caregivers. The questionnaire covered several domains, including individual characteristics, family and social factors, environmental conditions, and parenting practices.

Individual characteristics included child gender and the number of siblings. The number of siblings referred to the child’s biological siblings and was dichotomized as having at least one sibling or being an only child.

Family and socioeconomic factors included parental education level, household income, parental age at childbirth, primary caregiver, family structure, and parental separation. Parental education level was categorized into two groups: undergraduate education or below and postgraduate education (master’s degree or higher). Because the vast majority of parents had relatively high educational attainment and some lower educational categories contained very few participants, educational levels were consolidated for statistical analysis. Average household income was categorized based on monthly per capita income as <5,000,000 VND and ≥5,000,000 VND (approximately USD 200 at the time of the study). Parental age at childbirth was categorized as <31 years and ≥31 years. Parents as primary caregivers referred to children whose main daily caregiver was one or both parents. Family structure was classified as nuclear or extended family. Separation from mother or father was defined as the absence of the respective parent from the child’s daily living environment for at least 6 consecutive months.

Medical and perinatal factors included history of seizures, low birth weight (<2500 g), preterm birth (<37 weeks of gestation), cesarean delivery, and birth asphyxia, as reported by caregivers or obtained from medical records when available. Exclusive breastfeeding was defined as feeding the child only breast milk for the first six months of life.

Environmental and parenting practices included screen exposure, use of TV/mobile devices, language exposure, and caregiver–child interaction. Screen exposure was defined as the total cumulative daily duration of exposure to screen-based devices, including television, smartphones, tablets, and other electronic screens, as reported by the primary caregiver. The use of TV/mobile devices to calm or occupy the child referred to the use of screen-based devices to soothe the child during distress or to keep the child occupied without active interaction.

Listening to language audio was defined as exposure to audio-based language input in Vietnamese, such as songs, stories, or recorded speech. White noise exposure was defined as the use of continuous background sounds, typically low-frequency or natural ambient sounds (e.g., rainfall, flowing water, or rustling leaves), intended to facilitate sleep. Passive language exposure was defined as exposure to a non-native language (other than Vietnamese) through listening only, without direct interaction or structured instruction. Early bilingual exposure was defined as active exposure to a second language in addition to Vietnamese, including formal or informal contexts in which the child engages with the language.

Early talking with the child referred to the initiation of regular and intentional caregiver–child verbal interaction, such as talking, singing, naming objects, or responding to the child’s vocalizations. This variable was categorized according to whether such interactions were reported to have been initiated before 6 months of age or at/after 6 months of age. The 6-month threshold was selected because it corresponds to an important prelinguistic developmental period during which canonical babbling and increasingly interactive vocal communication typically emerge [13]. This categorization was intended to distinguish children who were exposed to regular caregiver verbal interaction during early infancy from those whose exposure began later. Shared reading or picture book viewing was defined as interactive reading or viewing activities between the child and caregiver.

### 2.5. Statistical Analysis

Statistical analyses were performed using SPSS software (version 20.0). Categorical variables were summarized as frequencies and percentages. Univariate logistic regression analyses were initially conducted to examine the association between each independent variable and language delay. Variables with a *p*-value < 0.25 in the univariate analyses were considered candidates for multivariable analysis and were subsequently entered into a multivariable logistic regression model. Logistic regression was selected because the outcome variable (language delay versus no language delay) was binary. Both the Enter and Backward Likelihood Ratio methods were applied to identify independent factors associated with language delay and to assess the robustness of the findings. Odds ratios (ORs) with 95% confidence intervals (CIs) were calculated, and a two-sided *p*-value < 0.05 was considered statistically significant.

## 3. Results

A total of 110 children were enrolled, including 55 children with language delay and 55 children with typical language development. The mean age was 29.51 ± 0.79 months in the language delay group and 27.31 ± 1.38 months in the control group, with no statistically significant difference between groups (*p* = 0.146). Sex distribution was also comparable between groups, with males accounting for 65.5% and 56.4% of participants in the language delay and control groups, respectively (*p* = 0.329). These findings indicate that the two groups were generally comparable with respect to baseline demographic characteristics (Table 1).

### 3.1. Univariate Logistic Regression

Table 2 presents the results of univariate logistic regression analyses for factors associated with language delay in children aged 12 to 36 months.

Univariate logistic regression analyses identified several factors associated with language delay in children aged 12–36 months. Some factors were protective, reducing the likelihood of delay, while others were associated with an increased risk.

Among the protective factors, children whose mothers were employed had a significantly lower risk of language delay than those whose mothers were unemployed (OR = 0.154; 95% CI: 0.042–0.568; *p* = 0.005). Similarly, listening to language audio (OR = 0.298; 95% CI: 0.136–0.655; *p* = 0.003), passive language exposure (OR = 0.410; 95% CI: 0.183–0.918; *p* = 0.030), and exposure to white noise (OR = 0.380; 95% CI: 0.148–0.977; *p* = 0.045) were associated with a reduced likelihood of language delay. Notably, shared reading or picture viewing emerged as the strongest protective factor (OR = 0.137; 95% CI: 0.043–0.436; *p* = 0.001). Early bilingual exposure was also identified as a significant protective factor (OR = 0.267; 95% CI: 0.089–0.796; *p* = 0.018).

Conversely, several factors were associated with an increased risk of language delay. The most prominent risk factor was the use of television or mobile devices to calm or occupy children, which increased the likelihood of language delay by approximately 29-fold (OR = 29.264; 95% CI: 10.117–84.646; *p* < 0.001). In addition, daily screen exposure exceeding 2 h was associated with an approximately fivefold increase in risk compared with limited screen use (OR = 4.929; 95% CI: 1.893–12.834; *p* = 0.001). Other variables, including child gender, parental education level, parental age at childbirth, obstetric history, birth weight, preterm birth, cesarean delivery, birth asphyxia, family structure, and parental separation, were not significantly associated with language delay (*p* > 0.05).

### 3.2. Multivariate Logistic Regression

Variables associated with language delay were initially screened at a significance level of *p* < 0.25 and subsequently included in a multivariable logistic regression model using the Enter method. The results of this model are presented in Table 3. The analysis revealed that only one variable—the use of television or mobile devices to calm or occupy children—was statistically significant (*p* < 0.001), with an odds ratio (OR) of 36.636. This finding indicates that children exposed to television or mobile devices for this purpose had approximately 36.6-fold higher odds of experiencing language delay than those who were not exposed. Other variables had *p* > 0.05 and were not statistically significant in the Enter model. However, shared reading or picture book viewing (*p* = 0.104) and early bilingual exposure (*p* = 0.105) showed near-significant trends, suggesting potential relevance. These variables may warrant further consideration in the Backward LR regression, as the elimination threshold for backward stepwise methods typically ranges between 0.10 and 0.20.

Variables associated with language delay at *p* < 0.25 were included in a multivariable logistic regression model and analyzed using the Backward Likelihood Ratio method. The final model is presented in Table 4. The selection process involved 16 sequential elimination steps, retaining only variables that remained statistically significant in the final model. The final model identified three factors significantly associated with language delay in children aged 12–36 months. Among these, the use of TV/mobile devices to calm or occupy the child was the strongest risk factor, increasing the odds of language delay by approximately 41-fold compared with the non-exposed group (OR = 41.344; 95% CI: 10.904–156.768; *p* < 0.001). Conversely, two protective factors were observed to reduce the risk of language delay: shared reading or picture book viewing (OR = 0.235; 95% CI: 0.055–1.005; *p* = 0.051) and early bilingual exposure (OR = 0.121; 95% CI: 0.023–0.650; *p* = 0.014). Other variables were not statistically significant after adjustment in the multivariable model, suggesting that their associations with language delay may be confounded or mediated by these key factors.

## 4. Discussion

Language delay is a common condition in early childhood, characterized by delayed achievement of age-appropriate language milestones. Between 12 and 36 months of age, children typically begin to build their basic vocabulary, combine words into short phrases, and express their needs and emotions verbally. When this process is delayed, children may experience long-term difficulties in communication, learning, and cognitive development [2]. Identifying risk and protective factors associated with language delay during this critical period is essential for early detection, timely intervention, and prevention.

In our study, among the 15 variables analyzed, three were significantly associated with language delay: the use of TV or mobile devices to calm or occupy the child, shared reading or picture book viewing, and early bilingual exposure. Among these, screen use for calming or occupying the child was the strongest risk factor, whereas the other two were protective factors.

Multivariable logistic regression revealed that children who were exposed to TV or mobile devices to calm or occupy them had 36.6-fold higher odds of language delay compared with those who were not exposed. This association increased to approximately 41-fold in the Backward Likelihood Ratio model (*p* < 0.001). Univariate analysis also showed that screen exposure exceeding two hours per day was associated with an increased risk of language delay (OR = 4.93; 95% CI: 1.89–12.83; *p* = 0.001). These findings strongly suggest a negative impact of screen exposure on early language development. Similarly, a study in Thailand reported that children who watched TV or digital media for more than two hours per day were 3.82 times more likely to have language delay compared with those with limited exposure [4]. A large-scale study in Denmark also confirmed this association, showing that daily digital device use of one hour or more significantly increased the risk of both receptive (AOR = 1.30–1.42) and expressive language delays (AOR = 1.19–1.46) [14].

In modern households, particularly in urban settings, the use of TV or smartphones to calm or occupy children has become increasingly common. Many parents, due to busy schedules or time constraints, rely on screen-based devices as a convenient means of managing children’s behavior. However, growing evidence suggests that early and prolonged exposure to electronic screens may adversely affect brain development, cognitive function, emotional regulation, and social skills. Screen use reduces opportunities for two-way verbal interaction between the child and caregiver, which is a critical component of early language acquisition. When children are exposed primarily to one-way visual and auditory input without reciprocal interaction, their ability to process sounds, comprehend meaning, and develop grammatical structures may be compromised. Previous studies have emphasized the importance of interactive communication in supporting language development [15]. In addition, some evidence suggests that electromagnetic emissions from electronic devices may affect neural function, potentially impairing attention and information processing [3]. Moreover, excessive screen time may displace essential developmental experiences, including physical activity, social interaction, and environmental exploration, all of which are important for language learning. According to the American Academy of Pediatrics, children under 18 months should avoid screen exposure (except for video calls), while those aged 2–5 years should be limited to less than one hour per day, preferably with parental interaction to promote comprehension and active engagement [16]. From this perspective, language acquisition is facilitated not merely by exposure to linguistic input but by contingent, responsive interactions that support joint attention, turn-taking, and meaning-making. Excessive screen use may reduce opportunities for such interactions, thereby limiting language-learning experiences during a critical developmental period.

In contrast, shared reading or picture book viewing was associated with a protective effect against language delay. Multivariable analysis showed an odds ratio of 0.235 (95% CI: 0.055–1.005; *p* = 0.051), suggesting a borderline protective association. Shared reading may promote language development through several complementary mechanisms. Beyond increasing children’s exposure to vocabulary and grammatical structures, book-sharing activities facilitate joint attention, conversational turn-taking, and scaffolding behaviors by caregivers. These interactions provide opportunities for children to connect words with meanings and contextual experiences, thereby strengthening both receptive and expressive language skills [17]. Importantly, the benefits of shared reading appear to depend more on the quality of caregiver–child interaction than on the format of the material.

While traditional print books are often considered the standard, shared picture viewing on digital devices (e.g., smartphones or tablets) may also support language development when it involves active engagement, such as labeling, questioning, and turn-taking. However, several studies suggest that screen-based formats may be less effective when they reduce interaction or introduce distractions (e.g., animations or passive viewing). Therefore, the protective effect observed in this study likely reflects interactive shared engagement rather than the specific medium used.

Our findings are consistent with previous studies. Children who were read to more than four times per week for over 10 min per session had a significantly lower likelihood of language delay (OR = 0.16; 95% CI: 0.03–0.72), whereas those who were not read to were more than twice as likely to experience language delay (PR = 2.38; *p* = 0.035) [18]. In contrast, children who were not read to were more than twice as likely to experience language delay (PR = 2.38; *p* = 0.035). Similarly, a meta-analysis synthesizing data from 19 randomized controlled trials (N = 2594) demonstrated that shared book reading significantly improved expressive language (d = 0.41) and receptive language (d = 0.26), and substantially enhanced caregiver communication skills (d = 1.01) [5]. These findings support shared reading as an effective early intervention for promoting language development and reducing the risk of delay.

Early bilingual exposure was also identified as a protective factor (OR = 0.121; 95% CI: 0.023–0.650; *p* = 0.014), indicating substantially lower odds of language delay among exposed children. However, the effect of bilingualism on language development remains debated. Historically, concerns have been raised that exposure to two languages during early childhood may delay language acquisition because children must learn and differentiate between two linguistic systems simultaneously. Some studies have reported that bilingual children may exhibit temporary delays in single-language expression; however, their total vocabulary across both languages is comparable to or even greater than that of monolingual peers [7,8]. Our findings are consistent with more recent research indicating that early bilingual exposure does not adversely affect language development and may, in some contexts, confer developmental advantages. One possible explanation is that bilingual environments often provide children with richer and more diverse linguistic input, increasing opportunities for communication across different social contexts. Exposure to multiple language systems may also promote metalinguistic awareness, cognitive flexibility, and attentional control, which have been proposed as mechanisms supporting both language learning and broader cognitive development [19]. The simultaneous activation of two linguistic systems may strengthen executive functioning and language-switching abilities. Taken together, these findings suggest that natural, interactive bilingual exposure supports rather than hinders language and cognitive development. In the context of increasing globalization, our results add to the growing body of evidence indicating that early bilingual exposure may serve as a facilitator, rather than a barrier, to optimal language development.

Although early talking with the child did not reach statistical significance in the final multivariable model, the observed trend toward a protective association is consistent with previous research highlighting the importance of caregiver-directed speech and responsive verbal interactions in supporting early language acquisition and vocabulary development. Regular verbal engagement between caregivers and children may provide rich linguistic input and opportunities for social communication during a critical period of language development.

In contrast, several demographics, perinatal, and family-related variables, including child sex, parental education, birth history, birth weight, family structure, and parental absence, were not significantly associated with language delay in the present study. Although these factors have been identified as potential determinants of language outcomes in some previous studies, their effects have been inconsistent across populations and developmental stages. One possible explanation is that during the first three years of life, proximal environmental factors–particularly those directly influencing the child’s daily communicative experiences–may exert a more immediate impact on language acquisition than broader demographic or socioeconomic characteristics. This interpretation is consistent with ecological and socio-interactionist models of language development, which emphasize the central role of responsive caregiver–child interactions and language-rich environments in shaping early communication skills. The absence of significant associations for several demographic and perinatal variables should also be interpreted cautiously. Previous studies have reported inconsistent findings regarding factors such as child sex, parental education, household income, birth weight, and preterm birth. While some investigations have identified these variables as risk factors for language delay, others have found limited or no independent associations after adjustment for environmental and interaction-related factors. In addition, the relatively modest sample size of the present study may have limited the statistical power to detect small-to-moderate associations for some variables. Therefore, the absence of statistical significance should not be interpreted as evidence that these factors are unimportant, but rather that their effects may be weaker, context-dependent, or influenced by other interacting variables. The present findings therefore suggest that modifiable interactional and environmental factors may be especially important targets for early identification and intervention efforts aimed at reducing the risk of language delay.

The present study contributes to the growing body of literature on language delay in several important ways. First, it provides empirical evidence from Vietnam, a lower-middle-income Southeast Asian country that remains underrepresented in research on early language development. Given that much of the existing evidence regarding factors associated with language delay has been derived from high-income Western countries, our findings extend the existing knowledge base to a Vietnamese context. From a theoretical perspective, the observed associations between screen use, shared reading, bilingual exposure, and language outcomes support the importance of environmental and interaction-based influences on early language development. Moreover, the results support contemporary ecological and multifactorial perspectives of language development. The identification of screen exposure as a risk factor, alongside the protective associations of shared reading and early bilingual exposure, underscores the importance of children’s early language environments. These findings are consistent with the view that rich and responsive communicative environments may facilitate language acquisition, whereas excessive passive screen exposure may limit opportunities for language learning. Furthermore, by focusing on children aged 12–36 months, the study contributes to a better understanding of language delay during a critical developmental period. While previous research has frequently focused on preschool- and school-aged children, the present findings highlight factors that may influence language outcomes before formal diagnosis is typically established, thereby providing insight into early language development and opportunities for prevention. Taken together, these findings contribute to current theoretical understandings of early language development and emphasize the potential role of modifiable environmental factors in shaping children’s language outcomes.

Several limitations of this study should be acknowledged. First, the case–control design allows the identification of associations but does not permit causal inferences. Therefore, the observed relationships between screen exposure, shared reading, early bilingual exposure, and language delay should be interpreted with caution. Prospective longitudinal studies are needed to further clarify the temporal and causal relationships between early environmental exposures and language development. Second, several variables were based on parental reports and may therefore be subject to recall bias and reporting inaccuracies. In addition, because all questionnaires and interviews were conducted in Vietnamese, parents or caregivers who were unable to adequately understand Vietnamese were excluded. This criterion may have limited the participation of some linguistically diverse families and introduced a degree of selection bias. Third, although the study achieved the calculated sample size, the relatively modest sample and recruitment from a single study setting may limit the generalizability of the findings to the broader population of Vietnamese children. In addition, the number of variables evaluated relative to the sample size may have affected the stability and precision of some multivariable estimates, as reflected by the wide confidence intervals observed for certain odds ratios. Therefore, the identified associations should be interpreted with caution and considered exploratory until confirmed by larger prospective studies. Future multicenter studies involving larger and more diverse populations across different regions of Vietnam are warranted to improve external validity. Fourth, the age range of 12–36 months encompasses a period of rapid language development, and the present study was not designed to evaluate age-specific effects. Future studies with larger samples should examine whether factors associated with language delay differ across narrower age groups. Finally, although a wide range of demographic, perinatal, and environmental factors were evaluated, residual confounding from unmeasured variables cannot be completely excluded. In addition, bilingual exposure was assessed as a dichotomous variable, and detailed information regarding the type of second language, the relative proportion of exposure to each language, and the context or intensity of bilingual exposure was not collected. Consequently, the association observed between bilingual exposure and language delay should be interpreted cautiously. Future research should incorporate more comprehensive measures of bilingual language input and explore additional developmental, familial, and environmental factors that may influence language outcomes in early childhood.

## 5. Conclusions

Our study identified several key factors associated with language delay in children aged 12–36 months. Among these, the use of television or mobile devices to calm or occupy children emerged as the strongest risk factor, substantially increasing the odds of language delay. In contrast, shared reading or picture book viewing and early bilingual exposure were identified as protective factors. These findings highlight the importance of a positive and interactive language environment between caregivers and children, which may play a more influential role in early language acquisition than demographic or socioeconomic factors. Practical interventions—such as limiting screen time, promoting shared reading and conversational activities, and encouraging play-based interactions—may help reduce the risk of language delay. Furthermore, there is a need to strengthen parental education and public health communication on evidence-based parenting practices, with a focus on early detection and timely intervention for language development disorders in young children.

## Figures and Tables

**Table 1:** Characteristics of the Study Participants and Comparison Between the Case and Control Groups.

Characteristic	Case Group (n = 55)	Control Group (n = 55)	*p*-Value
Age (months)	29.51 ± 0.79	27.31 ± 1.38	0.146
Gender			0.329
Male	36 (65.5%)	31 (56.4%)	
Female	19 (34.5%)	24 (43.6%)	

**Table 2:** Univariate logistic regression analysis of factors associated with language delay in children aged 12 to 36 months.

Independent Variable	B	OR	95% CI	*p*-Value
Male gender	0.383	1.467	0.679–3.168	0.329
Father’s education level	−0.087	0.917	0.406–2.074	0.835
Mother’s education level	0.747	2.111	0.890–5.006	0.090
Mother employed	−1.872	0.154	0.042–0.568	**0.005**
Average household income	0.613	1.846	0.751–4.536	0.181
Number of siblings	−0.538	0.584	0.269–1.266	0.173
Father’s age < 31	−0.221	0.802	0.377–1.703	0.565
Mother’s age < 31	−0.390	0.677	0.311–1.473	0.325
History of seizures	0.712	2.038	0.179–23.151	0.566
Low birth weight	−0.307	0.736	0.157–3.452	0.697
Preterm birth	−0.732	0.481	0.084–2.742	0.410
Cesarean section	−0.245	0.783	0.354–1.730	0.545
Birth asphyxia	0.000	1.000	0.061–16.401	1.000
Exclusive breastfeeding	−0.304	0.738	0.343–1.587	0.437
Breastfed exclusively	0.000	1.000	0.460–2.175	1.000
Screen exposure > 2 h/day	1.595	4.929	1.893–12.834	**0.001**
Listening to language audio	−1.210	0.298	0.136–0.655	**0.003**
Using white noise	−0.966	0.380	0.148–0.977	**0.045**
Passive language exposure	−0.892	0.410	0.183–0.918	**0.030**
Early talking with child	−0.865	0.421	0.174–1.017	0.055
Shared reading or picture book viewing	−1.986	0.137	0.043–0.436	**0.001**
Parents as primary caregivers	−0.696	0.498	0.190–1.308	0.157
Use of TV/mobile devices to calm or occupy the child	3.376	29.264	10.117–84.646	**0.000**
Nuclear family	0.602	1.826	0.882–3.779	0.105
Separation from mother	−0.127	0.880	0.327–2.369	0.801
Separation from father	0.523	1.687	0.787–3.617	0.179
Early bilingual exposure	−1.322	0.267	0.089–0.796	**0.018**

**Table 3:** Multivariate logistic regression (Enter method) results of factors associated with language delay in children aged 12 to 36 months.

Independent Variable	B	OR	95% CI	*p*-Value
Mother’s education level	0.108	1.114	0.181–6.852	0.907
Mother employed	−0.833	0.435	0.062–3.033	0.401
Average household income	0.057	1.058	0.148–7.567	0.955
Number of siblings	−0.513	0.599	0.056–6.418	0.672
Father’s age < 31	−1.085	0.338	0.034–3.338	0.353
Mother’s age < 31	−0.453	0.636	0.086–4.686	0.657
Screen exposure > 2 h/day	0.349	1.418	0.278–7.229	0.674
Listening to language audio	0.306	1.358	0.303–6.080	0.689
Using white noise	−0.167	0.846	0.160–4.489	0.845
Passive language exposure	−0.600	0.549	0.115–2.623	0.452
Early talking with child	−0.602	0.548	0.123–2.432	0.428
Shared reading or picture book viewing	−1.611	0.200	0.029–1.393	**0.104**
Parents as primary caregivers	−0.872	0.418	0.071–2.464	0.335
Use of TV/mobile devices to calm or occupy the child	3.601	36.636	7.998–167.816	**0.000**
Early bilingual exposure	−1.675	0.187	0.025–1.423	**0.105**

**Table 4:** Multivariate logistic regression (Backward LR method) results of factors associated with language delay in children aged 12 to 36 months.

Independent Variable	B	OR	95% CI	*p*-Value
Shared reading or picture book viewing	−1.447	0.235	0.055–1.005	0.051
Use of TV/mobile devices to calm or occupy the child	3.722	41.344	10.904–156.768	<0.0001
Early bilingual exposure	−2.109	0.121	0.023–0.650	0.014

## Data Availability

The data supporting the findings of this study are not publicly available due to privacy and ethical restrictions involving pediatric participants. De-identified data are available from the corresponding author upon reasonable request, subject to approval by the institutional ethics committee.
